# What’s New in Paediatric Sepsis

**DOI:** 10.1007/s40124-016-0093-4

**Published:** 2016-02-20

**Authors:** Deborah Farrell, Simon Nadel

**Affiliations:** St Mary’s Hospital and Imperial College London, Praed St, London, W2 1NY UK

**Keywords:** Paediatric, Sepsis, Shock, Treatment

## Abstract

Severe sepsis and septic shock remains a leading cause of mortality and morbidity in children. There is ongoing uncertainty regarding the optimal treatment pathways however the initial management of sepsis is crucial. This article is designed to be an informal and personal review of recent developments in paediatric sepsis over the past 3 years.

## Introduction

Severe sepsis and septic shock remains a leading cause of mortality and morbidity in children. Despite advances in prevention and treatment, children with severe sepsis continue to present significant treatment challenges to clinicians. Current treatment guidelines have been developed from the best available evidence ranging from multiple large randomized controlled trials to consensus of expert opinion, often extrapolating from adult data.

Research is ongoing in many aspects of sepsis in both the adult and paediatric population.

This article is designed to be an informal and personal review of recent developments in paediatric sepsis over the past 3 years.

## Current Experience

Over the last 10 years or so, the epidemiology of paediatric severe sepsis has been dramatically affected by the introduction of effective vaccines against what were the most common causes of community-acquired sepsis in children.

Beginning with the introduction of the HiB (Haemophilus influenzae type B) vaccine into the routine schedule of infant vaccines in the early 1990s, and subsequent introduction of the Men C (serogroup C meningococcal) vaccine and then 7- and 13-valent pneumococcal vaccines and the recent introduction of the Men B (serogroup B meningococcal) vaccine, the whole pattern of admissions to PICU with severe sepsis has completely changed.

It is becoming increasingly common that patients admitted to paediatric intensive care with severe sepsis in this current post-vaccine era are those with extreme prematurity, pre-existing co-morbidities or who are immunocompromised in some way [1•].

Because of this change in epidemiology, understanding the pathophysiology of paediatric sepsis has proven more difficult, and rational interventions to interrupt the “sepsis cascade” have proven less effective than predicted, probably because these children are not previously healthy with community-acquired sepsis, but are a heterogeneous mixture of patients with varied pathologies.

It is likely that more generic measures or “bundles of care”, such as are recommended in tools such as the “Surviving Sepsis Campaign”, will prove beneficial in paediatric practice, as they have been shown to be in adult practice [2••].

## Surviving Sepsis Campaign

Current guidelines regarding the treatment of sepsis have been dominated by successive Surviving Sepsis Campaign (SSC) publications, the latest in 2012 [2••]. These recommendations have been developed with significant multi-national representation and cooperation. The recommendations are the product of systematic reviews of the evidence and expert opinion following extensive literature review.

The surviving sepsis campaign recommendations focus on early identification of severe sepsis and septic shock, prompt administration of IV antibiotics with haemodynamic resuscitation and then ongoing supportive care.

Paediatric recommendations differ from the adult recommendations where there is specific evidence relating to children available or where it is felt that evidence from adult studies would be inappropriate to extrapolate. The Paediatric Considerations in the campaign differ from the adult guidance on several issues. These include recommendations in children for the use of intraosseous access and peripheral vasoactive infusions when there is difficulty in gaining central venous access; the rapidity of fluid resuscitation; the type and dose of vasoactive infusions and the use of lung protective ventilation strategies.

The American College of Critical Care Medicine has also published Clinical Guidelines for Hemodynamic Support of Neonates and Children with Septic Shock (ACCM) [3••]. These have the advantage of being specific for the neonatal and paediatric populations, and many of their recommendations have been adopted by the Surviving Sepsis Campaign.

Both the SSC and ACCM guidelines are currently being updated.

## New Findings

### Early Goal Directed Therapy

The identification of physiological targets in the immediate management of patients with sepsis and septic shock has been labelled “Early Goal Directed Therapy”. The first reliable evidence that targeting blood pressure, perfusion (urine output) and central venous oxygen saturation was beneficial to outcome (as determined by mortality) was published in a landmark paper by Rivers et al. [4]. This was a single-centre randomized, proof-of-concept trial which showed that hospital mortality and length of hospital stay are improved in patients with septic shock who are resuscitated according to protocol-driven goal directed therapy. The goals differed from standard goals by targeting central venous oxygen saturation in addition to other more readily available parameters. While early goal directed therapy was readily adopted into Surviving Sepsis Campaign guidelines, there remained controversy regarding the external validity of the original trial, the difficulty of implementation and potential complications of certain aspects of the protocol. These controversies are particularly relevant to the paediatric population, especially in regard to the need to obtain early central venous access in the superior thoracic distribution (internal jugular or subclavian veins) for the measurement of central venous oxygen saturation.

Three major trials concerning goal directed therapy have been published in the past year in the New England Journal of Medicine. These trials were undertaken across multiple centres in the United States (ARISE), England (ProMISE) and Australasia (ProCESS) [5••, 6••, 7••]. In total, over 4000 adult patients were enrolled in these trials comparing early goal directed therapy to usual care. The ProCESS trial also included a third arm of Protocol-Based Standard therapy. Each trial independently found that the patients who received early goal directed therapy had no significant benefit in mortality, organ dysfunction or intensive care stay compared with usual care or protocol-based care (Table 1). It is important to state that in the 13 years since the Rivers study was published, there have been huge advances in the delivery of protocolised care to patients with sepsis and septic shock, due to the broad uptake of the SSC guidelines [8•]. This suggests that routine care for these patients has dramatically improved, with consequent improvement in outcomes, and suggests that despite good evidence for the use of a central venous oxygen saturation target, the benefit of achieving such a target is not accompanied by demonstrable improvements in outcome when compared with current standard care.

**Table 1:** Compiled mortality data of ProCESS, ProMISE and ACCESS trials

Any cause day 90 mortality—no/total no (%)	EGDT	Usual care	Protocol-based standard therapy
ProMISE	184/623 (29.5)	181/620 (29.2)	–
ARISE	147/792 (18.6)	150/796 (18.8)	–
ProCESS	129/405 (31.9)	139/412 (33.7)	128/415 (30.8)

Whilst there were no paediatric patients enrolled in these trials, they provide compelling evidence that goal directed therapy in sepsis does not provide additional benefit over usual care. A surrogate for measurement of central venous oxygen saturation is measurement of whole blood lactate. While this gives a measure of global oxygen utilization, several studies have demonstrated the utility of measuring lactate clearance as a marker of adequacy of resuscitation and association with outcome, in both adults and children [9–11].

## Timing of Antibiotics

A long-held tenet of the treatment of septic shock has been the early administration of empiric antibiotics.

A large international multicentre trial looking at almost 18,000 adult patients with severe sepsis or septic shock demonstrated a significant correlation between delay in antibiotic administration and mortality, increased illness severity and organ dysfunction [12••]. This study demonstrated that the odds ratio for death increased steadily each hour antibiotics were delayed (see Table 2). This is a timely confirmation that the timing of antibiotic therapy is still paramount once septic shock and severe sepsis has been recognized. A recent paediatric study has demonstrated a similar correlation between delayed antibiotic administration and risk of organ dysfunction [13•].

**Table 2:** Hospital mortality odds ratios

Time to antibiotics	Odds ratio	95 % Confidence interval	*P* value
0–1	1.00		
1–2	1.07	0.97–1.18	0.165
2–3	1.14	1.02–1.26	0.021
3–4	1.19	1.04–1.35	0.009
4–5	1.24	1.06–1.45	0.006
5–6	1.47	1.22–1.76	<0.001
>6	1.52	1.36–1.70	<0.001

### Fluid Resuscitation

Following the publication of the Fluid Expansion as Supportive Therapy (FEAST) Trial in 2011, which demonstrated increased mortality in children with presumed sepsis treated with fluid bolus therapy compared with maintenance fluid, there has been intense interest in the possible disadvantages of fluid overload in the treatment of paediatric septic shock [14]. Given the population and setting of the FEAST study, it is unclear how relevant this study is to resource-rich settings.

Further rigorous and well-powered research is required prior to any change in the current consensus. There is no doubt that early, aggressive fluid resuscitation has been associated with the improvements in outcome of severe sepsis and septic shock that have been demonstrated [3••, 15]. Although rigorously conducted randomized controlled trials are difficult to conduct in these circumstances, the recent adult studies referred to above suggest that such studies are possible, and this should be the priority in paediatric practice. It is heartening that such a study, in pilot form, has recently been funded by the UK National Institute of Health Research [16].

## Steroids

The use of steroids in sepsis has been an ongoing debate in clinical practice and international literature. There are pathophysiological reasons, including sepsis-associated adrenal suppression, inotrope unresponsiveness and exaggerated inflammatory responses, which logically lead to the conclusion that corticosteroid therapy should provide multiple benefits to patients with shock. However, the evidence base does not reflect this theory in practice.

There has been conflicting evidence regarding the benefits of steroid use in adults with shock and no large randomized controlled trials in a paediatric population. A meta-analysis published in 2013 concluded that there is very limited evidence to support the use of steroids in paediatric septic shock [17•]. There is a considerable need for well-designed and appropriately powered trials in resource-rich settings to fully delineate the possible benefits of steroid therapy, particularly in children.

## Future Directions

Aside from trials looking into already established treatments, there is also considerable interest in determining what patient factors increase a child’s risk of a serious sepsis event. Research into these patient characteristics remains exploratory but provides an exciting opportunity for the future development of novel treatments or therapeutic approaches.

## Genetics

There has been an explosion in the development of technology to allow research into the impact of genetics on the susceptibility to, and severity of infections. Numerous studies have evaluated gene polymorphisms in both the adult and paediatric population with severe sepsis. Many of these studies have demonstrated associations between disease severity and presence of various polymorphisms. However, most of these studies have suffered from being underpowered, having heterogenous populations and not being able to validate the preliminary results [18].

The use of Genome-Wide studies has become possible in the last 5 years, and further developments in technology will undoubtedly provide the possibility of offering personalized therapies [19]. Current technology allows the dissection of genetic pathways within hours of sampling, and this offers the possibility of intervening specifically to influence particular pathways which have been activated or suppressed in individual patients [20•].

Currently, this research does not yet have clinical applications however in the future genetic testing may also be useful in patient risk stratification.

## Vitamin D

Vitamin D has been investigated in the context of several conditions including asthma, eczema, respiratory tract infections and sepsis.

There have been recent studies demonstrating associations between Vitamin D levels and severity of sepsis. These studies have shown that children with low Vitamin D levels were more likely to have sepsis, increased illness severity and spend longer in PICU [21, 22•]. There is some discussion around whether Vitamin D levels taken in children with septic shock after significant fluid resuscitation are reflective of their premorbid levels. A recent meta-analysis in adults demonstrated similar findings [23].

The next stage of research impacting on treatment guidelines would be an assessment of whether treatment of Vitamin D deficiency whilst critically unwell reduces PICU stay and improves prognosis.

## Conclusion

Over the past 3 years, sepsis research has focused on initial fluid resuscitation and goal directed therapy. There is an ongoing need to extrapolate adult data to the paediatric population. However, further research in a paediatric setting is required to develop the optimal approach to resuscitation. The main focus of ongoing research is focused on genetic studies, which may lead to interesting new strategies. The central tenets of paediatric sepsis management remain constant with the confirmation of benefit of early antibiotic administration and appropriate supportive therapy as advised by international guidelines.
